# E3 ubiquitin ligase SYVN1 is a key positive regulator for GSDMD-mediated pyroptosis

**DOI:** 10.1038/s41419-022-04553-x

**Published:** 2022-02-03

**Authors:** Yuhua Shi, Yang Yang, Weilv Xu, Dongyun Shi, Wei Xu, Xinyu Fu, Qian Lv, Jie Xia, Fushan Shi

**Affiliations:** 1 Department of Veterinary Medicine, College of Animal Sciences, Zhejiang University, Hangzhou, 310058 Zhejiang PR China; 2 Zhejiang Provincial Key Laboratory of Preventive Veterinary Medicine, Zhejiang University, Hangzhou, 310058 Zhejiang PR China; 3 Key Laboratory of Applied Technology on Green-Eco-Healthy Animal Husbandry of Zhejiang Province, Zhejiang Provincial Engineering Laboratory for Animal Health Inspection & Internet Technology, College of Animal Science and Technology & College of Veterinary Medicine of Zhejiang A&F University, Hangzhou, 311300 Zhejiang China

**Keywords:** Cell death and immune response, Immune cell death

## Abstract

Gasdermin D (GSDMD) participates in the activation of inflammasomes and pyroptosis. Meanwhile, ubiquitination strictly regulates inflammatory responses. However, how ubiquitination regulates Gasdermin D activity is not well understood. In this study, we show that pyroptosis triggered by Gasdermin D is regulated through ubiquitination. Specifically, SYVN1, an E3 ubiquitin ligase of gasdermin D, promotes GSDMD-mediated pyroptosis. SYVN1 deficiency inhibits pyroptosis and subsequent LDH release and PI uptake. SYVN1 directly interacts with GSDMD, and mediates K27-linked polyubiquitination of GSDMD on K203 and K204 residues, promoting GSDMD-induced pyroptotic cell death. Thus, our findings revealed the essential role of SYVN1 in GSDMD-mediated pyroptosis. Overall, GSDMD ubiquitination is a potential therapeutic module for inflammatory diseases.

## Introduction

Pyroptosis is a form of programmed cell death, characterized by cell swelling, pore formation in the cell membrane, and cell lysis, releasing the cytoplasmic contents [1–4]. It plays an important role in host defense and inflammatory responses [5]. Recent studies show that pyroptosis is induced by proteolytic cleavage of Gasdermin D (GSDMD) by inflammatory caspases [2, 6, 7]. Canonical inflammasomes, including NLRP3 inflammasome, activate caspase-1. Meanwhile, lipopolysaccharide (LPS) activates noncanonical inflammasomes via the caspase-11 in mice or caspase-4 and -5 in humans [8–12]. As the final downstream effector of inflammasomes activation, GSDMD is cleaved by inflammatory caspases at the junction between the N-terminal cytotoxic domain (GSDMD-p30) and C-terminal autoinhibitory domain (GSDMD-p20) [5, 13, 14]. GSDMD-p30 then binds (cellular) phospholipids and oligomerizes to form 10–20 nm pores on the plasma membrane, triggering pyroptotic cell death [15–19].

Posttranslational modification of inflammasome components via ubiquitin (Ub) is critical for the regulation of inflammasomes activation [20, 21]. Ubiquitination of NLRP3, caspase-1/11, ASC, IL-1β, and other inflammasome components regulates several essential nodes in the regulatory networks [20–27]. E3 ubiquitin ligases such as Pellino 2 mediates K63-linked polyubiquitination and NLRP3 inflammasome activation [28]. In addition, HUWE1 stimulates inflammasomes activation by promoting K27-linked polyubiquitination of NLRP3, AIM2, and NLRC4, which strengthen host defense against bacterial infection [29]. Gasdermin B (GSDMB), another member of the gasdermin family, has also been recently found to participate in ubiquitination-mediated pyroptosis that blocks bactericidal functions of NK cells [30]. Given that pyroptosis regulation is important in the signaling of cell death, GSDMD ubiquitination may broadly be thought to regulate the function of GSDMD and the activities of different inflammasomes [31]. Importantly, the function and mechanisms underlying posttranslational modifications of GSDMD, such as ubiquitination, deubiquitination, and phosphorylation, are still unknown.

Synoviolin (SYVN1), also known as Hrd1, is one of the RING E3 ligases [32]. Given that SYVN1 targets numerous substrates such as a proapoptotic factor (IRE1) [33], B lymphocyte–induced maturation protein 1 (BLIMP1) [34], and mitochondrial antiviral signaling (MAVS) [35], it performs distinct functions in different cells. SYVN1 regulates ER-stress-induced cell death by promoting ubiquitination and degradation of IRE1. Moreover, SYVN1 promotes T-cell immunity [36], B-cell immunity [37] and regulates TLR-induced inflammation through K27-linked ubiquitination and inactivation of Usp15 [38]. However, the role of SYVN1 in pyroptosis is not well understood.

In this study, we found that SYVN1 promotes canonical and noncanonical inflammasome-induced pyroptosis through promoting GSDMD ubiquitination. Particularly, SYVN1 induces GSDMD-mediated pyroptotic cell death by promoting K27-linked polyubiquitination of GSDMD on K203 and K204 residues. Our findings revealed a new mechanism of regulating pyroptotic cell death through posttranslational modification of GSDMD.

## Results

### Ubiquitination of GSDMD

**Fig. 1:**
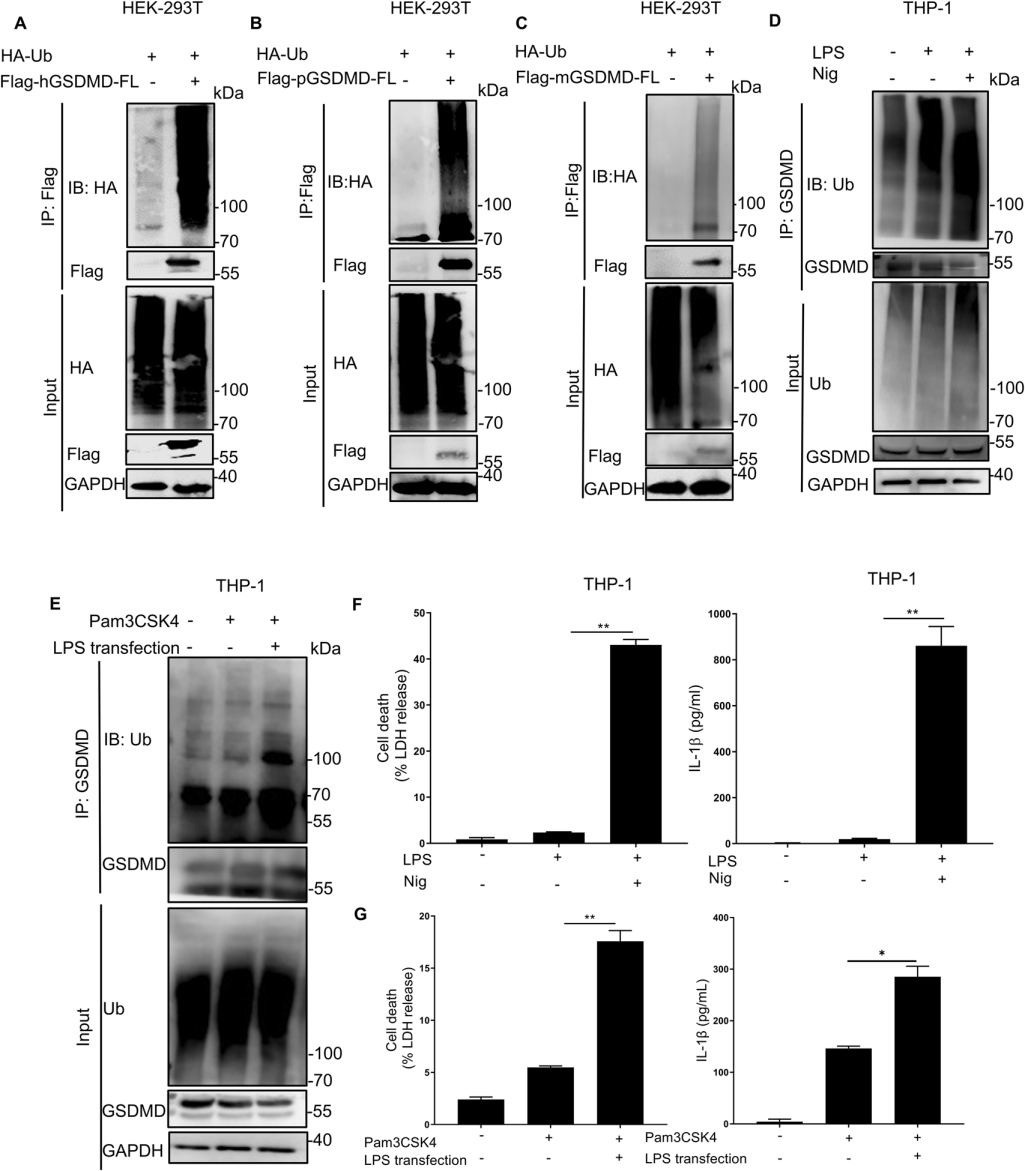
GSDMD can be modified by ubiquitination. **A** Lysates from HEK293T cells co-transfected with HA-Ub along with Flag-human GSDMD (hGSDMD) or not were subjected to immunoprecipitation with Flag antibody followed by immunoblotting using an anti-HA antibody. **B** Immunoprecipitation and immunoblotting of HEK293T cells co-transfected with HA-Ub and Flag-porcine GSDMD (pGSDMD) using anti-Flag and anti-HA antibodies, respectively. **C** Immunoprecipitation and immunoblotting of HEK293T cells co-transfected with HA-Ub and Flag-mouse GSDMD (mGSDMD) using anti-Flag and anti-HA antibodies, respectively. **D**, **F** THP-1 cells were primed with LPS (500 ng/ml) for 4 h, and then treated with or without Nigericin (10 μM). Cell lysates were immunoprecipitated with anti-GSDMD antibody, followed by immunoblotting with anti-GSDMD and anti-Ub antibodies, respectively. Secretion of IL-1β was analyzed using ELISA. LDH release was analyzed using an LDH release assay. **E**, **G** THP-1 cells were incubated with Pam3CSK4 (500 ng/ml) for 4 h, and then transfected with or without LPS (2 μg/ml) for 6 h. Cell lysates were immunoprecipitated with anti-GSDMD antibody, followed by immunoblotting with anti-GSDMD and anti-Ub antibodies. IL-1β secretion in THP-1 cells was analyzed using ELISA, whereas LDH release was analyzed using LDH release assay. All results shown are representative of at least three independent experiments.

Prior to this study, GSDMD ubiquitination and its effect on pyroptosis have not been reported. Therefore, we first transfected HEK293T cells with plasmids encoding HA-ubiquitin and Flag-GSDMD to test whether GSDMD can be ubiquitinated. Immunoprecipitation was performed using an anti-Flag antibody, whereas immunoblotting was performed using anti-HA or anti-Flag. We found that co-expression of HA-ubiquitin and Flag-human GSDMD-induced ubiquitination of human GSDMD (Fig. 1A). Further studies confirmed that both murine and porcine GSDMD proteins can also be ubiquitinated (Fig. 1B, C). In addition, LPS/nigericin or Pam3CSK4 treatment followed by LPS transfection, which activated canonical and noncanonical inflammasomes, substantially increased ubiquitination of endogenous GSDMD in THP-1 cells (Fig. 1D–G). Accordingly, we hypothesized that GSDMD ubiquitination is a critical and efficiently regulated process.

### Identification of SYVN1 as a GSDMD-interacting E3 ligase

**Fig. 2:**
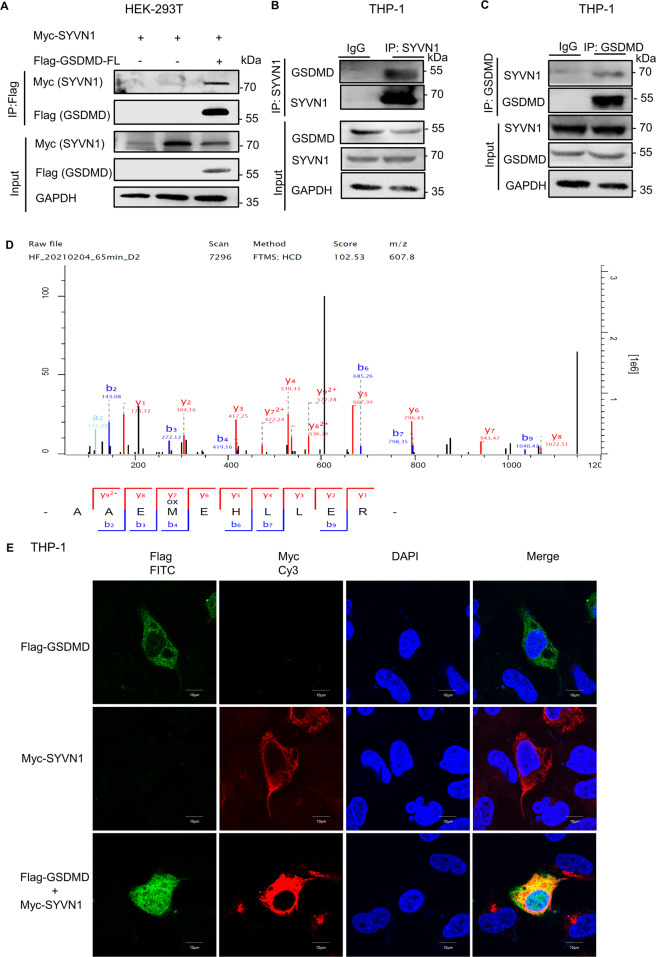
SYVN1 interacts with endogenous and exogenous GSDMD. **A** HEK293T cells co-transfected with pcDNA3.1-SYVN1-Myc and p3 × Flag-GSDMD were lysed and immunoprecipitated with anti-Flag antibody. Both the immunoprecipitates (IP) and whole-cell lysates (Input) were subjected to gel electrophoresis and analyzed by immunoblotting with anti-Myc and anti-Flag antibodies, respectively. **B**, **C** Immunoprecipitation analysis of THP-1 cells using anti-SYVN1 or anti-GSDMD antibodies. Immunoblotting of both IP and total proteins in THP-1 cells were performed using anti-SYVN1 and anti-GSDMD antibodies, respectively. **D** HEK293T cells co-transfected with pcDNA3.1-SYVN1-Myc and p3 × Flag-GSDMD were lysed and immunoprecipitated with anti-Flag antibody. The potential GSDMD-binding proteins in HEK293T cells were evaluated using Co-IP and MS analysis. The MS/MS spectrum of 71-AAEMEHLLER-80 is shown. Observed b- and y-ion series are indicated. **E** Immunostaining analysis of HEK293T cells after transfection with pcDNA3.1-SYVN1-Myc and p3 × Flag-GSDMD using anti-Flag and anti-Myc antibodies. Subcellular localization of Flag-GSDMD (green), Myc-SYVN1 (red), and DAPI (blue, a nucleus marker) were observed using confocal microscopy. Scale: 1 bar represents 10 μm. All experiments shown are representative of at least three independent experiments.

Since E3 ligase plays an indispensable role in the ubiquitination process, we explored the involvement of this enzyme in GSDMD ubiquitination. Analysis of the UbiBrowser database revealed that SYVN1 is one of the most important E3 ligases involved in GSDMD ubiquitination (Supplementary Fig. 1A). HEK293T cells were then transfected with Myc-tagged SYVN1 and Flag-GSDMD. Co-immunoprecipitation (Co-IP) assay showed that GSDMD interacted with SYVN1 (Fig. 2A). IP‐LC‐MS/MS analysis revealed comparable findings (Fig. 2D and Supplementary Fig. 1B). Further analysis revealed that endogenous GSDMD also interacted with SYVN1 in THP-1 cells (Fig. 2B, C). Indirect immunofluorescence further confirmed that the interaction between SYVN1 and human GSDMD occurred in the cytoplasm (Fig. 2E). Therefore, SYVN1 interacts with both endogenous and exogenous GSDMD.

Given that GSDMD is the key effector of pyroptosis downstream of activated canonical and noncanonical inflammasomes [5, 6], we evaluated the role of GSDMD ubiquitination in pyroptosis. Since HEK293T cells do not express endogenous GSDMD [39, 40], we reconstructed canonical and noncanonical pyroptosis in HEK293T cells. Expression of both GSDMD and caspase-1/4 significantly induced LDH production (Supplementary Fig. 2A, B). Immunoblotting revealed that caspase-1 and caspase-4 cleaved the full-length GSDMD into active N-terminal GSDMD-p30 domain (Supplementary Fig. 2A, B), and obvious propidium iodide (PI) staining was observed by fluorescence microscopy (Supplementary Fig. 2C, D). The same results were observed in LDH and PI staining of GSDMD-p30 triggered pyroptosis (Supplementary Fig. 2A–D). However, the expression of GSDMD-p30 was not detectable, in agreement with the hypothesis that the protein might have a toxic effect on the host cells [41]. Overall, these results show that the canonical and noncanonical inflammasome-mediated pyroptosis were successfully reconstructed in vitro.

### SYVN1 promotes GSDMD-mediated pyroptosis

**Fig. 3:**
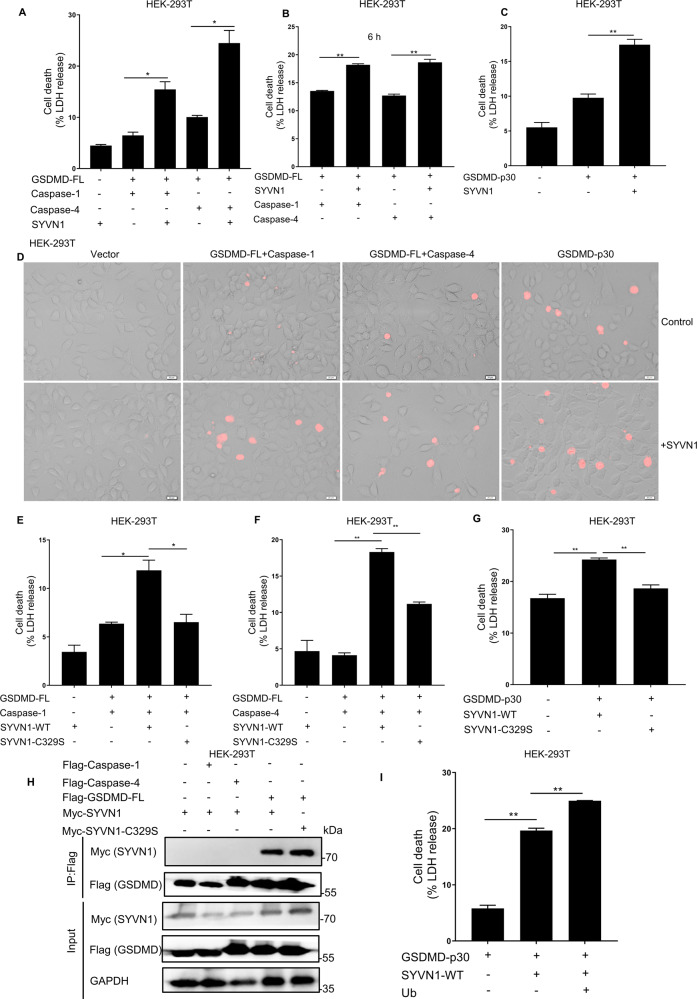
Overexpression of SYVN1 promotes GSDMD-triggered pyroptosis. **A**, **D** HEK293T cells were transfected with pCMV-Myc-Caspase-1/4 (100 ng), p3 × Flag-hGSDMD-FL (200 ng), and p3×Flag-SYVN1 (600 ng) or control vector. The supernatants were collected and analyzed by LDH release assay and cell staining with PI after 24 h transfection. PI analysis was performed using 2.5 μg/ml. Scale: 1 bar represents 20 μm. **B** HEK293T cells were transfected with Myc-SYVN1 (600 ng) and p3 × Flag-hGSDMD-FL (200 ng) for 12 h and then transfected with caspase-1/4 (100 ng) for 6 h. **C**, **D** HEK293T cells were transfected with Myc-SYVN1 (600 ng) and hGSDMD-p30-Myc (100 ng). Supernatants were collected and analyzed by LDH release assay and cell staining with PI at 24 h after transfection. **E**, **F** LDH release assay of HEK293T cells after co-transfection with caspase-1/4 (100 ng), hGSDMD-FL (200 ng), and SYVN1-WT (600 ng) or SYVN1-C329S (600 ng). **G** LDH releases assay of HEK293T cells co-transfected with hGSDMD-p30 (100 ng) and SYVN1-WT (600 ng) or SYVN1-C329S (600 ng). The supernatants were collected and analyzed by LDH release assay at 24 h after transfection. **H** Immunoprecipitation (IP) and immunoblot (IB) analysis of HEK293T cells co-transfected with pcDNA3.1-SYVN1-Myc and p3 × Flag-hGSDMD-FL or p3 × Flag-Caspase-1/4. IP was performed using an anti-Flag antibody, whereas IB was performed using an anti-Myc antibody. **I** LDH release of HEK293T cells co-transfected with hGSDMD-p30, SYVN1, and Ub. The analysis was performed 24 h after transfection. Data were representative of three independent experiments.

To determine whether SYVN1 regulates GSDMD-mediated pyroptosis, we evaluated LDH release and PI staining (red) based on the reconstruction of canonical and noncanonical pyroptosis models in HEK293T cells. SYVN1 overexpression markedly increased canonical and noncanonical pyroptosis when co-expressing caspase-1/4 and GSDMD (Fig. 3A, D and Supplementary Fig. 3A). To confirm that SYVN1 performed its function independent of caspase-1/4, caspase-1/4 was transfected into HEK293T cells 12 h after GSDMD and SYVN1 co-transfection. LDH release and PI staining were significantly increased compared to the group without SYVN1 transfection (Fig. 3B and Supplementary Fig. 3B, C). In addition, SYVN1 interacted with GSDMD but not caspase-1/4 (Fig. 3H). Accordingly, we speculated that SYVN1 promotes pyroptosis by directly targeting GSDMD. To validate this hypothesis, we evaluated whether SYVN1 directly targets GSDMD N-terminal (GSDMD-p30) to promote pyroptosis. Of note, transfection of SYVN1 with GSDMD-p30 markedly enhanced LDH release and PI uptake compared to the group without SYVN1 transfection (Fig. 3C, D). These results demonstrated that SYVN1 promotes pyroptosis mainly by targeting the GSDMD-p30 fragment. To further investigate whether the E3 ubiquitin ligase activity of SYVN1 is responsible for regulating GSDMD-induced pyroptosis, we assessed the effects of SYVN1 and SYVN1-C329S on pyroptosis in HEK293T cells. Based on previous reports, the cysteine residue at position 329 is critical, and its mutation to serine (C329S) abolishes SYVN1 E3 activity [38, 42]. The results showed that SYVN1-C329S could not increase LDH release (Fig. 3E–G). To determine whether GSDMD ubiquitination facilitates pyroptosis, HEK293T cells were co-transfected with GSDMD-p30 and wild-type Ub. It was found that overexpression of Ub significantly promoted GSDMD-p30-mediated pyroptosis (Supplementary Fig. 3D, E), which indicates that ubiquitination of GSDMD facilitates pyroptosis. Importantly, the addition of Ub further enhanced SYVN1 promoted GSDMD-p30-mediated pyroptosis (Fig. 3I). Collectively, these findings demonstrated that SYVN1 promotes pyroptosis through the ubiquitination of GSDMD.

### Inhibition of SYVN1 impairs GSDMD-mediated pyroptosis

**Fig. 4:**
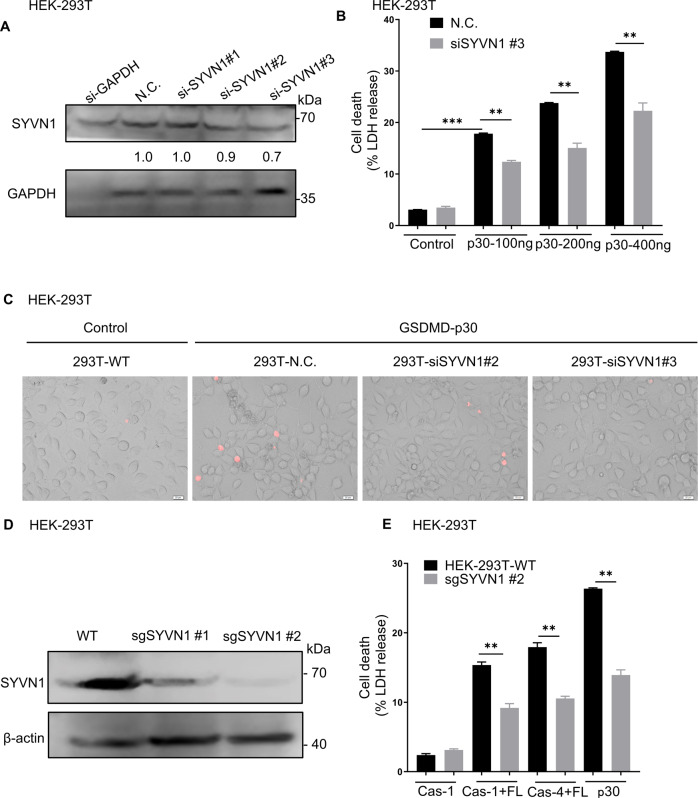
Effect of inhibiting SYVN1 on GSDMD-mediated pyroptosis in HEK293T cells. **A** Immunoblotting analysis for efficiency of SYVN1 knockdown in HEK293T cells using siSYVN1. The analysis was performed using an anti-SYVN1 antibody. **B**, **C** HEK293T cells were transfected siSYVN1-control (50 nM) or siSYVN1 (50 nM), 48 h after transfection, cells were transfected with hGSDMD-p30. The supernatants were collected and analyzed by LDH release assay. After 24 h transfection, the cells were stained with PI (2.5 μg/ml) and analyzed by fluorescence microscopy. **D** The efficiency of SYVN1 knockout was analyzed by immunoblotting with anti-SYVN1 antibody in HEK293T cells obtained from WT and SYVN1-KO HEK293T cells. **E** HEK293T WT and SYVN1-KO cells were transfected with caspase-1/4 and hGSDMD-FL or hGSDMD-p30 alone. The supernatants were collected and analyzed by LDH release assay. All results are representative of at least three independent experiments.

To further confirm the role of SYVN1 in pyroptosis, we designed three siRNAs targeting SYVN1. The efficiency of SYVN1 knockdown was shown in Fig. 4A, and we ultimately selected siSYVN1 #3 for further studies because of its better knockdown efficacy (Fig. 4A). LDH assay and PI uptake revealed that SYVN1 knockdown inhibited the effect of GSDMD-p30 on pyroptosis in HEK293T cells (Fig. 4B, C and Supplementary Fig. 4). To further investigate the role of SYVN1 in pyroptosis, we generated SYVN1 knockout HEK293T cells by CRISPR/Cas9-mediated genome editing (according to reference [43]). The efficiency of SYVN1 knockout was shown in Fig. 4D. Consistent with the results of knockdown of SYVN1, knockout of SYVN1 in HEK293T cells significantly suppressed canonical, noncanonical inflammasomes and GSDMD-p30-mediated pyroptosis (Fig. 4E). Collectively, these findings further demonstrated that SYVN1 promotes GSDMD-mediated pyroptosis.

**Fig. 5:**
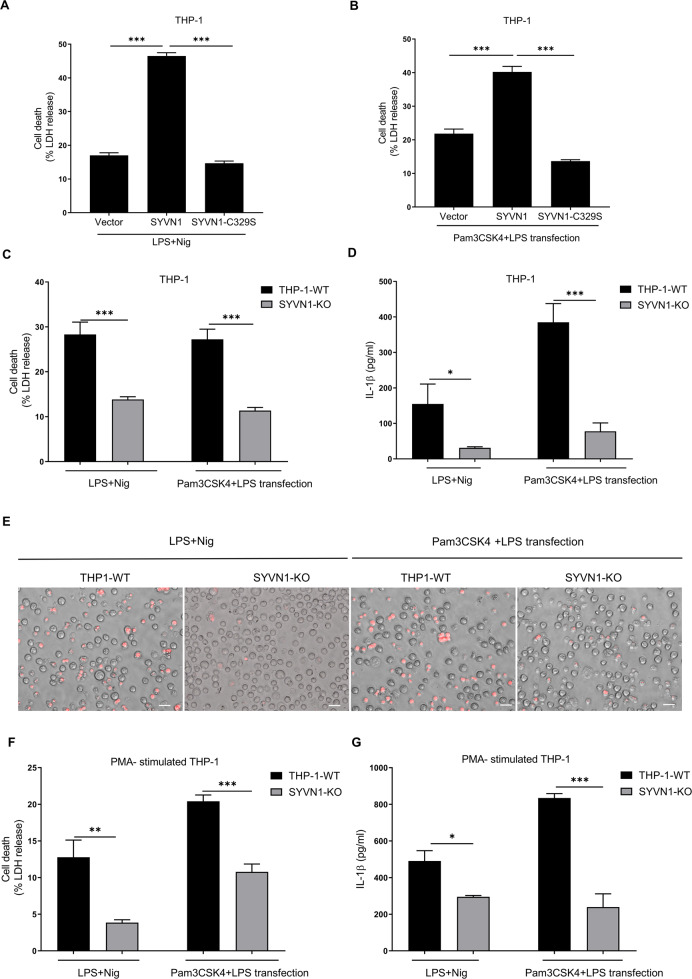
Effect of inhibiting SYVN1 on pyroptosis in THP-1 cells. **A** THP-1 cells electrotransfected with SYVN1 or SYVN1-C329S for 24 h were incubated with 500 ng/mL LPS for 4 h and then another 1 h for 10 μM Nigericin. The supernatants were collected and analyzed by LDH release assay. **B** THP-1 cells electrotransfected with SYVN1 or SYVN1-C329S were incubated with 500 ng/mL Pam3CSK4 for 4 h and then transfected with 2 μg/ml LPS for 6 h. The supernatants were collected and analyzed by LDH release assay. **C**–**E** THP-1 WT and SYVN1-KO cells were stimulated with LPS and Nigericin or stimulated with Pam3CSK4 and transfected with LPS the same way as above. The supernatants were collected and analyzed by LDH release assay (**C**) and ELISA for IL-1β (**D**). The cells were observed by using PI staining (**E**). Scale: 1 bar represents 20 μm. **F**, **G** PMA-primed THP-1 WT, and SYVN1-KO cells were stimulated with LPS and Nigericin or stimulated with Pam3CSK4 and transfected with LPS the same way as above. The supernatants were collected and analyzed by LDH release assay (**F**) and ELISA for IL-1β (**G**). Data were representative of three independent experiments.

To test the effect of SYVN1 on pyroptosis in THP-1 cells. THP-1 cells were first electroporated with plasmid encoding SYVN1 or SYVN1-C329S (SYVN1 mutant, which lacks E3 ligase activity), and then stimulated with canonical and noncanonical inflammasomes stimuli. We noticed that the secretion of LDH induced by LPS + Nigericin or Pam3CSK4 + LPS transfection was further remarkably enhanced by the SYVN1 overexpression (Fig. 5A, B). However, SYVN1-C329S failed to increase the secretion of LDH (Fig. 5A, B). To determine the effect of SYVN1 deficiency on the pyroptosis in THP-1 cells, we generated SYVN1 knockout THP-1 cells by CRISPR/Cas9-mediated genome editing. SYVN1 knockout significantly decreased the pyroptosis induced by canonical and noncanonical inflammasomes stimuli (Fig. 5C–E). In addition, SYVN1 knockout macrophages, which differentiated from THP-1 stimulated by phorbol-12-myristate-13-acetate (PMA), showed similar results (Fig. 5F, G). Thus, SYVN1 plays a key role in pyroptosis induced by canonical and noncanonical inflammasomes activation in THP-1 cells.

### SYVN1 ubiquitinates GSDMD with K27-linked polyubiquitin chains

**Fig. 6:**
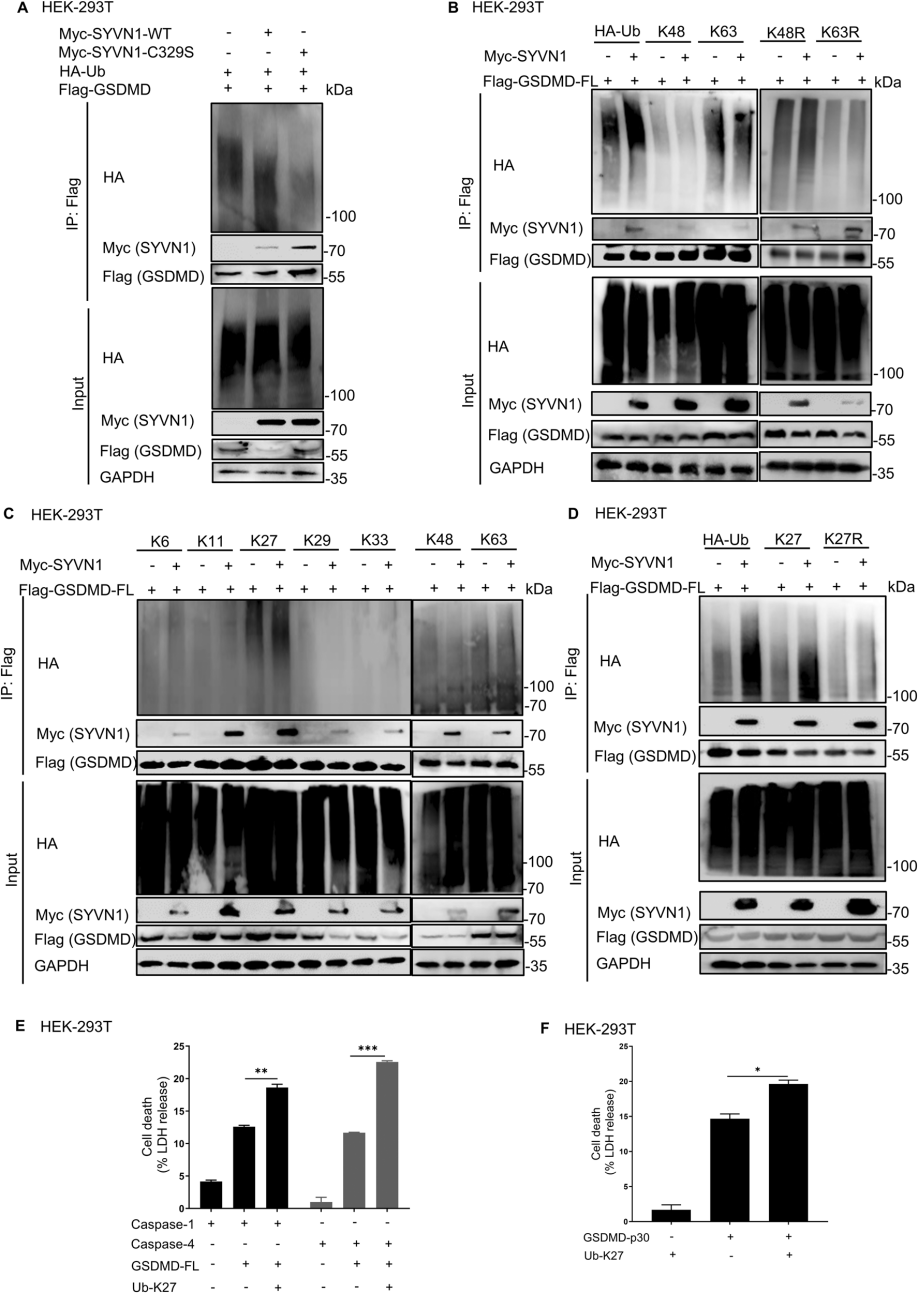
SYVN1 ubiquitinates GSDMD with K27-linked polyubiquitin chains. **A** Immunoprecipitation analysis of HEK293T cells co-expressing Flag-GSDMD and HA-Ub together with Myc-SYVN1 or Myc-SYVN1-C329S. Anti-Flag immunoprecipitates were analyzed using immunoblotting with indicated antibodies. The expression levels of the transfected proteins were analyzed using immunoblotting with indicated antibodies. **B** Immunoprecipitation analysis of HEK293T cells co-expressing Flag-GSDMD and Myc-SYVN1 together with HA-Ub (K48, K63, K48R, or K63R) as indicated. Immunoblotting analysis was performed using anti-Myc or anti-Flag antibodies. Expression of the transfected proteins was analyzed using immunoblotting, using corresponding antibodies. **C** Immunoprecipitation analysis of HEK293T cells expressing Flag-GSDMD and Myc-SYVN1 together with HA-Ub (K6, K11, K27, K29, K33, K48, or K63 only) as indicated. Anti-Flag immunoprecipitates were analyzed using immunoblotting with anti-Myc or anti-Flag antibodies. Expression of the transfected proteins was analyzed using immunoblotting, using corresponding antibodies. **D** Immunoprecipitation analysis of HEK293T cells expressing Flag-GSDMD and Myc-SYVN1 together with HA-Ub (K27 or K27R) as indicated. Anti-Flag immunoprecipitates were analyzed using immunoblotting with indicated antibodies. Expression of the transfected proteins was analyzed using immunoblotting, using corresponding antibodies. **E** LDH release assay of HEK293T cells transfected with caspase-1/4, GSDMD and Ub-K27 or control vector. **F** LDH release assay of HEK293T cells transfected with hGSDMD-p30 and Ub-K27 or control vector. All data were representative of three independent experiments.

Considering that SYVN1 regulates pyroptosis in an E3 ligase activity-dependent manner, we speculated SYVN1 mediates polyubiquitination of GSDMD. As such, we co-expressed Myc-SYVN1-WT or the E3 ligase-dead mutant SYVN1-C329S with HA-ubiquitin (HA-Ub) and Flag-GSDMD in HEK293T cells. Compared to C329S mutant, overexpression of SYVN1-WT substantially increased GSDMD ubiquitination (Fig. 6A), suggesting that E3 ubiquitin ligase activity of SYVN1 is necessary for GSDMD ubiquitination. Since K48 and K63 are the most studied branched chains, we further investigated the role of these two polyubiquitination chains in the ubiquitination of GSDMD. HEK293T cells were transfected with Flag-GSDMD and HA-K48-ubiquitin, HA-K63-ubiquitin, HA-K48R-ubiquitin, or HA-K63R-ubiquitin. The results showed that SYVN1 had no effect on K48 and K63-mediated polyubiquitination of GSDMD (Fig. 6B). Also, SYVN1 promoted K48R and K63R-mediated polyubiquitination, suggesting that SYVN1 induced the addition of other ubiquitination chains on GSDMD. Given that seven lysine residues (K6, K11, K27, K29, K33, K48, and K63) have been reported to form polyubiquitin chains [44–48], we sequentially co-transfected these HA-tagged ubiquitin mutants (with only one of the seven lysine residues retained as lysine, and the other six replaced with arginine) with Flag-tagged GSDMD with or without SYVN1 into HEK293T cells. The co-immunoprecipitation assay revealed that K27 ubiquitin mutant markedly increased polyubiquitination of GSDMD (Fig. 6C). However, overexpression of SYVN1 had no effect on GSDMD polyubiquitination in K27R mutant transfected cells (Fig. 6D). These findings suggest that SYVN1 promotes GSDMD-mediated pyroptosis through K27-linked polyubiquitination of GSDMD. Indeed, overexpression of Ub-K27 promoted pyroptosis induced by GSDMD-p30 and caspase-1/4-mediated GSDMD cleavage (Fig. 6E, F). Thus, SYVN1 mediates K27-linked polyubiquitination of GSDMD.

### K203 and K204 residues of GSDMD are polyubiquitinated by SYVN1

**Fig. 7:**
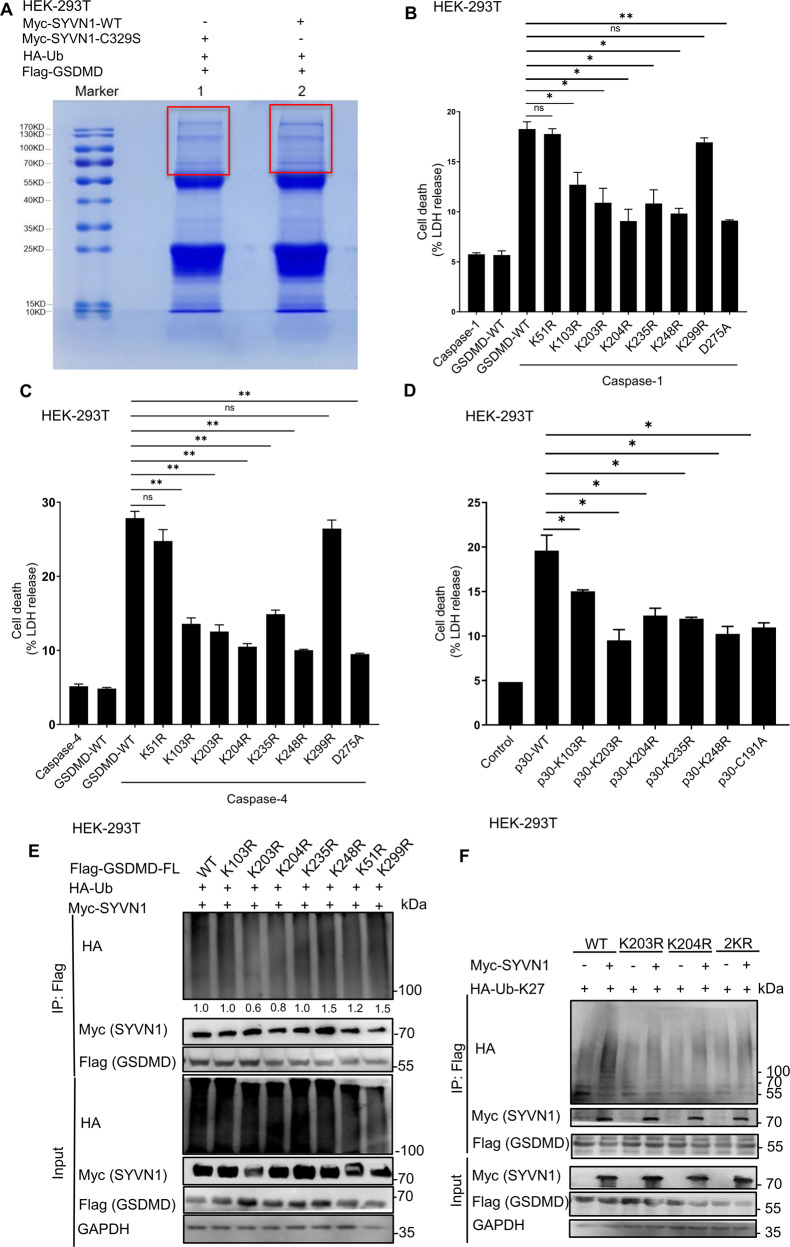
SYVN1 polyubiquitinates K203 and K204 residues of GSDMD. **A** Coomassie blue-stained bands of polyubiquitinated GSDMD. HEK293T cells were co-transfected with Flag-GSDMD, Myc-SYVN1 or Myc-SYVN1-C329S, and HA-ubiquitin (shown above the lanes). The square areas indicate regions from which proteins were removed for MS. **B**, **C** LDH assay of HEK293T cells transfected with pCMV-Myc-Caspase-1/4 (300 ng), p3 × Flag-hGSDMD-FL (600 ng), or GSDMD mutants (600 ng). The analysis was performed 24 h after transfection. **D** LDH releases assay of HEK293T cells transfected with GSDMD-p30 (300 ng) or GSDMD-p30 mutants (300 ng). The analysis was performed 24 h after transfection. **E** Immunoprecipitation analysis of HEK293T cells expressing Flag-GSDMD or Flag-GSDMD mutants together with HA-Ub and Myc-SYVN1. Expression of proteins of interest was analyzed using immunoblotting, using corresponding antibodies. Densitometry of the blots was measured using Image J. **F** Immunoprecipitation analysis of HEK293T cells expressing Flag-GSDMD, K203R, K204R, or 2KR mutants along with HA-Ub-K27 and Myc-SYVN1. Immunoblotting was performed using corresponding antibodies. All results are representative of three independent experiments.

To determine the sites of GSDMD polyubiquitination, HEK293T cells were co-transfected with Flag-GSDMD, HA-ubiquitin, and SYVN1-Myc or SYVN1-C329S-Myc. After immunoprecipitating GSDMD with anti-Flag purification beads, the proteins were separated using SDS-PAGE and visualized after Coomassie blue staining (Fig. 7A). To analyze ubiquitinated GSDMD sites by mass spectrometry (MS), the gel regions (Fig. 7A) which might contain ubiquitin-modified proteins were excised, digested with trypsin, and analyzed by liquid chromatography-MS. A database search of the MS/MS spectra revealed that GSDMD is the predominant protein identified in the samples. Representative spectra demonstrated that the K51 and K299 residues of GSDMD in the gel were modified with ubiquitin (Supplementary Fig. 5A). We also used the UbPred program (http://www.ubpred.org) [49–51], a predictor of potential ubiquitin-binding sites in proteins to predict the potential ubiquitination sites of GSDMD. This analysis revealed other five lysine residues: K103, K203, K204, K235, and K248 (Supplementary Fig. 5B). We then generated Flag-GSDMD-K51R, K103R, K203R, K204R, K235R, K248R, and K299R mutants in which each of these lysine residues was replaced with arginine (R). To determine if these potential ubiquitination sites altered pyroptotic function, HEK293T cells were co-transfected with wild-type GSDMD or GSDMD mutants and caspase-1/4. LDH release and pore formation assay revealed that pyroptosis significantly decreased in GSDMD-K103R, GSDMD-K203R, GSDMD-K204R, GSDMD-K235R, and GSDMD-K248R mutants, but not in GSDMD-K51R and K299R mutants (Fig. 7B, C and Supplementary Fig. 6A). GSDMD-D275A, which is the only cleavage site of caspase-1/4, was used as the positive control [5, 13]. As expected, caspase-1/4 did not cleave GSDMD-D275A, and the mutant (GSDMD-D275A) could not induce pyroptosis (Fig. 7B, C). Moreover, mutations in GSDMD-p30 ubiquitination sites substantially decreased GSDMD-p30-mediated pyroptosis (Fig. 7D and Supplementary Fig. 6B). GSDMD-p30-C191A was used as a positive control, given that mutation (C191A) in this amino acid decreases GSDMD-p30 oligomerization and inhibits pyroptosis [40, 52, 53]. Our results suggest that the amino acids K103, K203, K204, K235, and K248 are critical for GSDMD-mediated pyroptosis.

To further explore SYVN1-mediated ubiquitination sites on GSDMD, HEK293T cells were co-transfected with Myc-tagged SYVN1, HA-tagged ubiquitin, and Flag-tagged GSDMD or GSDMD K/R mutants. Compared to the WT GSDMD (Fig. 7E, lane 1), SYVN1 had no effect on ubiquitination of GSDMD-K51R or K299R mutant (Fig. 7E, lane 7, 8). In contrast, even though LDH decreased in GSDMD-K103R, K235R, and K248R mutants (Fig. 7B, C), SYVN1-mediated ubiquitination of GSDMD occurred in them (Fig. 7E, lane 2, 5, 6). Specifically, the Co-IP assay revealed that SYVN1-mediated GSDMD ubiquitination was partially blocked in K203R and K204R mutants (Fig. 7E, lane 3, 4). We further found that mutations in these two residues (2KR) did not completely prevent GSDMD ubiquitination (Fig. 7F), suggesting that SYVN1 also modifies other lysine residues. Overall, the above findings indicate that SYVN1 mainly ubiquitinates GSDMD at K203 and K204 residues, but there are also other lysine residues in GSDMD that can be ubiquitinated by SYVN1.

## Discussion

**Fig. 8:**
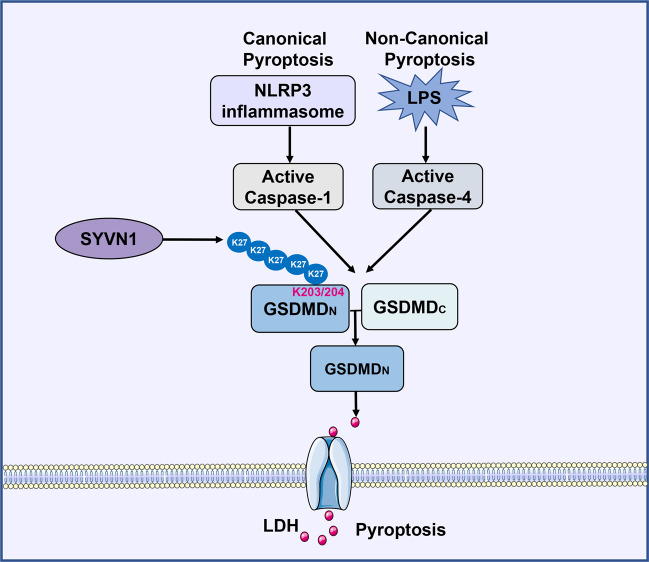
Diagram of SYVN1 regulating GSDMD-mediated pyroptosis. Working model for SYVN1 promoting the activation of canonical and noncanonical inflammasomes-mediated pyroptosis. SYVN1 binds to GSDMD, promotes the K27-linked polyubiquitination of K203 and K204 lysine residues on GSDMD, resulting in the increasement of pyroptosis.

Pyroptosis, mediated by the canonical and noncanonical inflammasomes, participates in the clearance of intracellular pathogens [6, 54]. Recent studies have identified Gasdermin D (GSDMD) as the primary executioner of pyroptosis [5, 6]. However, ubiquitination-mediated regulation of GSDMD activity is poorly understood. In this study, we demonstrated that ubiquitination of GSDMD plays a critical role in pyroptosis. Moreover, SYVN1 is an important E3 ubiquitin ligase that positively regulates canonical and noncanonical inflammasomes-mediated pyroptosis through K27-linked polyubiquitination of K203 and K204 lysine residues on GSDMD (Fig. 8).

Polyubiquitination is a posttranslational modification process that plays critical role in programmed cell death or NLRP3-mediated inflammasome activation through covalent linkage of ubiquitin to lysine residues in target proteins [20, 22, 55, 56]. Recent studies have demonstrated that GSDMD is essential in pyroptosis. Thus, it is reasonable to hypothesize that polyubiquitination of the common component of GSDMD effectively regulates pyroptosis in response to infection and danger signals. Herein, we demonstrated that ubiquitination of GSDMD facilitates GSDMD-mediated pyroptosis.

SYVN1 is over-expressed in synoviocytes of patients with rheumatoid arthritis, exacerbating the pathogenesis of the disease [57]. Moreover, SYVN1 regulates the functioning of immune cells, including T cells [36], B cells [37], and macrophages [38]. However, whether SYVN1 regulates pyroptosis had not been investigated. We found that overexpression of SYVN1 increases pyroptosis (Fig. 3), and as such, loss of SYVN1 function impairs GSDMD-mediated pyroptosis (Figs. 4, 5). In general, SYVN1 promotes GSDMD-mediated pyroptosis.

There were multiple K residues in GSDMD that can be ubiquitinated based on the prediction by Ubipred online software. These residues could be ubiquitinated by SYVN1 and other E3 ubiquitin ligase enzymes, and further studies are needed to identify these ubiquitin ligases. Herein, we found that SYVN1 promotes pyroptosis mainly by interacting and ubiquitinating K203 and K204 residues in GSDMD. The mass spectrometric analysis further revealed two high-confidence ubiquitin-modified residues in the N- (K51) and C-terminal (K299) domains of GSDMD (Supplementary Fig. 5A). However, a mutation in these residues (K51R/K299R) had no significant effect on GSDMD ubiquitination, suggesting that SYVN1 also modifies other lysine residues. A recent study revealed that pyroptotic cell death is independent of GSDMD-K204T modification [58]. In contrast, we found that GSDMD-K204R mutant inhibited GSDMD-induced pyroptosis. The discrepancy may result from the presence of threonine residue, which could also be ubiquitinated [59]. Further studies are needed to explore how mutations in GSDMD ubiquitination sites affect pyroptosis in vivo.

In conclusion, our study elucidated the unique role of GSDMD polyubiquitination in pyroptosis, in particular, mediated by SYVN1. First, we found that GSDMD ubiquitination occurs in several species including human, mouse, and swine. Second, SYVN1, an E3 ubiquitin ligase, facilitates GSDMD-mediated pyroptosis by promoting ubiquitination of GSDMD. Specifically, SYVN1 directly interacts with GSDMD and mediates K27-linked polyubiquitination of GSDMD on K203 and K204 residues to promote pyroptotic cell death. Thus, our results demonstrate that GSDMD ubiquitination may be an appealing target for the design of inflammatory diseases.

## Materials and methods

### Reagents

Anti-Flag M2 (F1804), anti-Flag M2 Magnetic Beads, anti-Myc (M5546), and anti-LPS (O11:B4) were purchased from Sigma-Aldrich. Anti-GSDMD (sc-393581) and anti-GAPDH (sc-47724) were from Santa Cruz Biotechnology. Anti-Ub (3936), anti-HA (3724), anti-β-actin (3700), and the secondary antibodies used for immunoblotting and immunofluorescence assay were purchased from Cell Signaling Technology. Anti-SYVN1 (ab170901) was purchased from Abcam, whereas Nigericin, Pam3CSK4 (tlrl-pms) were purchased from InvivoGen. ELISA kits for analysis of human IL-1β were purchased from MultiSciences. CytoTox 96 LDH-release assay kit (G1780) was purchased from Promega.

### Cell culture and stimulation

Human HEK293T cells were cultured in Dulbecco’s modified Eagle’s medium (DMEM) in a humidified incubator at 37 °C with 5% CO_2_, supplemented with 10% FBS (Corille, C1015-05), 100 μg/ml streptomycin, and 100 U/ml penicillin (Hyclone, 347 SV30010). Human monocyte cell line THP-1 was cultured in PRIM 1640 medium supplemented with 10% FBS, 100 μg/ml streptomycin, and 100 U/ml penicillin. To activate canonical inflammasomes, 5 × 10^5^ cells were plated overnight in 24-well plates and primed for 4 h with 500 ng/ml LPS. The cells were then stimulated for 1 h using 10 μM Nigericin. For noncanonical inflammasome activation, cells were primed for 4 h with 500 ng/ml Pam3CSK4, after which the medium was replaced and cells were transfected with 2 μg/ml LPS using Lipofectamine 2000 for 6 h.

### Plasmid construction and transfection

cDNAs for transfection in plasmids were obtained by reverse transcription of total RNA from HEK293T and THP-1 cells. The cDNAs were amplified using specific primers before sub-cloning the PCR products into p3 × Flag-CMV-7.1, pCMV-Myc, and pCDNA3.1 vectors. HA-tagged ubiquitin was provided by Dr. Yimin Yang (Zhejiang University). The primers used in this study are shown in Supplementary Table 1A.

### Induction of pyroptosis in HEK293T cells

HEK293T cells grown in 24-well plates to 80% confluence were transfected with 600 ng of p3×Flag-GSDMD and 600 ng of pCMV-Caspase-1/4 using VigoFect. The supernatants were collected 24 h after transfection and analyzed for IL-1β level using ELISA.

### ELISA

Supernatants from transfected cells were assayed for human IL-1β in accordance with the manufacturer’s instructions. Each experiment was performed independently at least three times. Sample preparation for ELISA was according to the method [60].

### Cytotoxicity assay and propidium iodide staining

The culture medium of HEK293T cells co-transfected for 24 h with p3 × Flag-CMV-7.1, pCMV-Myc, and pCDNA3.1 was collected and assayed for LDH release using the Cytotoxicity Detection Kit (Promega) according to the manufacturer’s manual. The HEK293T cells were cultured for 15 min with 2.5 μg/ml Propidium iodide (BD Bioscience, 556463) and observed under fluorescence microscopy. The experiments were performed independently in triplicate.

### Immunoblotting

Cells were lysed using lysis buffer (Beyotime, P0013) supplemented with Phenylmethanesulfonyl fluoride (PMSF) (Beyotime, ST506). Total proteins in the cells were extracted and thereafter separated using 12% SDS-PAGE. The proteins were then transferred onto the PVDF membrane, hybridized with primary antibodies, and probed with HRP-conjugated secondary antibodies. Proteins were detected using the image system (Clinx Science Instruments, China).

### Co-immunoprecipitation assay

Cells were lysed for 30 min in ice-cold lysis buffer and centrifuged at 10,000 × *g* at 4 °C. The supernatants were then incubated with anti-Flag binding beads (Sigma, M8823) at 4 °C. The beads were then washed three–five times with cold TBS. Immune complexes were denatured for 10 min at 100 °C in 1× SDS-PAGE loading buffer before immunoblotting analysis.

### Confocal immunofluorescence assay

HEK293T cells were seeded in 24-well at the rate of 1 × 10^5^ per well and transfected for 24 h with p3 × Flag-CMV-7.1, pCMV-Myc, and pCDNA3.1 plasmids. The cells were then fixed for 30 min at room temperature with 4% paraformaldehyde (Beyotime, P0098) before incubation with primary antibodies in DAPI (Beyotime, C1002). The cells were then observed using a laser scanning microscope (Olympus, IX81-FV1000).

### SYVN1 knockdown using small interfering RNA

Short interfering RNAs (Genepharma) specific for human SYVN1 were transfected into THP-1 and HEK293T cells using the Lipofectamine lipo8000 reagent (Beyotime, C0533FT) according to the manufacturer’s instructions. The sequences of the siRNAs used in this study are shown in Supplementary Table 1B.

### Construction of SYVN1 knockout cells using CRISPR/Cas9 technique

SYVN1 knockout HEK293T cells were generated using the CRISPR/Cas9 technique. Vectors expressing gRNA targeting human SYVN1 were transfected into HEK293T cells using the manufacturer’s protocol. In general, based on flow-cytometric analysis of GFP levels, SYVN1 knockout was achieved in 30–50% of HEK293T cells. For SYVN1 knockout THP-1 cells, gRNA plasmid was co-transfected with the lentiviral packaging vectors pMD2G and PSPAX2, then introduced into HEK293T cells to produce lentivirus. After 48 h, the viral supernatant was collected and added to THP-1 cells in six-well plates with a medium containing 8 μg/ml polybrene. The infected cells were spun at 500×*g* for 60 min, and fresh media was added. After 2 days’ infection, stably transfected cells were selected with GFP by flow-cytometric analysis. Single-cell sorting of transfected cells was performed using flow cytometry (MOFLO XDP). gRNA sequences are shown in Supplementary Table 1C.

### Transfection by electroporation of THP-1 cells

Electrotransfection experiments of THP-1 cells were performed using the 4D-Nucleofector™ X Unit (Lonza), following the manufacturer’s protocol. Briefly, 0.8 μg of each of the prepared plasmids were mixed with 100 μl 4D-Nucleofector™ Solution and co-transfected into 1 × 10^6^ THP-1 cells using the FF-100 program. After transfection, cells were transferred to a complete culture medium, followed by incubation for 24 h recovery in the 37 °C incubator. Cells were then harvested and seeded on 96-well plates at a density of 1 × 10^5^ cells/well for stimulation.

### LC-MS/MS analysis

Flag-tagged GSDMD immunoprecipitates prepared from whole-cell lysates or gel-filtrated fractions were resolved on SDS-PAGE gels, and protein bands were excised. The samples were digested with trypsin, and then subject to LC-MS/MS analysis. Swissprot_Human mass spectra were used as the standard reference. Trypsin/P was used for cleavage. MS data were captured and analyzed using Micrometer Biotech and Maxquant, respectively.

### Statistical analysis

The values are presented as mean ± SD. Data were analyzed using GraphPad Prism 8.0. The difference between experimental groups was assessed using Student’s *t*-test or one-way ANOVA followed by Tukey’s multiple comparisons. All experiments were performed independently at least three times. Statistical significance was set at *P* < 0.05, 0.01, or 0.001. *P represents *P* < 0.05, **P represents *P* < 0.01, and ***P represents *P* < 0.001.

## Supplementary information

Supplementary figure and table legends

Supplemental figure 1

Supplemental figure 2

Supplemental figure 3

Supplemental figure 4

Supplemental figure 5

Supplemental figure 6

Supplemental Table1

Author Contribution Statement

checklist

Author Agreement

## Data Availability

All data included in this study are available upon request by contact with the corresponding author.
