# Supplementary material for: E3 ubiquitin ligase SYVN1 is a key positive regulator for GSDMD-mediated pyroptosis
Source: Cell Death Dis. 2022 Feb 3;13(2):106. doi: 10.1038/s41419-022-04553-x (PMC8814081; doi:10.1038/s41419-022-04553-x)
Supplement: Supplementary file 1 — Supplementary figure and table legends [file 41419_2022_4553_MOESM1_ESM.docx]

**Supplementary Fig. 1 A,** Prediction of GSDMD ubiquitination by E3 ubiquitin ligase using the Ubibrowser software. **B,** MS/MS analysis for representative sequences of human SYVN1 alleles.

**Supplementary Fig. 2 Reconstruction of canonical and non-canonical inflammasomes-induced pyroptosis *in vitro*. A,** HEK293T cells were co-transfected with pCMV-Myc-Caspase-1 (600 ng) and p3×Flag-hGSDMD-FL (600 ng). pcDNA3.1-hGSDMD-p30-Myc (600 ng) was used as a positive control. Supernatants were analyzed using LDH assay. Cell lysates were normalized for proteins contents and analyzed using immunoblotting using antibodies specific for Flag, Myc and GAPDH. **B,** HEK293T cells were co-transfected with pCMV-Myc-Caspase-4 and p3×Flag-hGSDMD-FL. The pcDNA3.1-hGSDMD-p30-Myc was used as positive controls. Anti-Flag, Myc and GAPDH antibodies were used for immunoblotting. **C-D,** Pyroptosis of HEK293T cells after GSDMD cleavage as observed under bright-field and epifluorescent microscopy. Scale: 1 bar represents 100 μm. All results are representative of at least three independent experiments.

**Supplementary Fig. 3 A,** Bright-field and epifluorescent microscopy of HEK293T cells after PI staining. Scale: 1 bar represents 50 μm. **B,** HEK293T cells were transfected with Myc-SYVN1 and p3×Flag-hGSDMD-FL and thereafter for 18 h or 24 h with caspase-1/4. **C,** HEK293T cell were transfected with plasmids as shown. PI analysis was performed after 24 h transfection. Scale: 1 bar represents 50 μm. **D-E,**  HEK293T cells transfected with varied dose of hGSDMD-p30 and Ub. The supernatants were collected and analyzed after 24 h transfection for LDH release assay (D). HEK293T cells were observed under bright-field and epifluorescent microscopy after PI staining (E). Scale: 1 bar represents 100 μm.

**Supplementary Fig. 4** HEK293T cells under fluorescence microscopy after PI staining. The cells were first transfected with siSYVN1 (50 nM) or corresponding control and thereafter with hGSDMD-p30 after 48 h. Scale: 1 bar represents 50 μm.

**Supplementary Fig. 5** **Putative sites on hGSDMD**. **A,** Representative MS/MS spectra of ubiquitinated hGSDMD peptides. Protein samples were recovered from the gel, enzymatically digested and analyzed using Mass Spectrometry (MS). **B,** Ubiquitination patterns of GSDMD. Red areas represent ubiquitination sites predicted by Ubibrowser software, whereas the green sites represent ubiquitination sites identified by MS.

**Supplementary Fig. 6** **A-B,** PI staining analysis of HEK293T cells after 24 h transfection with indicated plasmids. Scale: 1 bar represents 50 μm.

**Supplementary Table 1** Primers used in this study.
