# Supplementary material for: E3 ubiquitin ligase SYVN1 is a key positive regulator for GSDMD-mediated pyroptosis
Source: Cell Death Dis. 2022 Feb 3;13(2):106. doi: 10.1038/s41419-022-04553-x (PMC8814081; doi:10.1038/s41419-022-04553-x)
Supplement: Supplementary file 9 — Author Contribution Statement [file 41419_2022_4553_MOESM9_ESM.docx]

# Author Contribution Statement

**MANUSCRIPT TITLE:** E3 ubiquitin ligase SYVN1 is a key positive regulator for GSDMD-mediated pyroptosis

**CONFLICT OF INTEREST:** The authors declare that they have no conflict of interest.

**AUTHOR CONTRIBUTIONS:** Yuhua Shi and WeiLv Xu performed most of experiments and contributed in writing the manuscript. Dongyun Shi, Wei Xu, Xinyu Fu, Qian Lv, Jie Xia performed some experiments. Yang Yang contributed in data analysis. Fushan Shi conceived the overall scope of the project and wrote the manuscript.



Author signature：


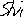

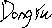

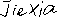

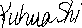

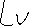

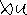

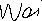

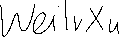

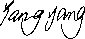

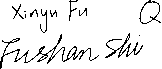

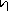

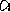

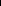


Date: 2021.12.20
