# Supplementary material for: E3 ubiquitin ligase SYVN1 is a key positive regulator for GSDMD-mediated pyroptosis
Source: Cell Death Dis. 2022 Feb 3;13(2):106. doi: 10.1038/s41419-022-04553-x (PMC8814081; doi:10.1038/s41419-022-04553-x)
Supplement: Supplementary file 11 — Author Agreement [file 41419_2022_4553_MOESM11_ESM.pdf]

首页 | 浙江大学邮件系统

https://mail.zju.edu.cn/coremail/XT5/index.jsp?sid=DAYKtZnMaOkQvZnBHNmQfSfGMrXNMiav#mail.read%7C%7B"fid"%3A1%2C"mid"%3A"1%3A1tbiAQIEA1ZdwoWoOgABsb"%2C"mboxa"%3A""%2C"start"%3A6%7D

浙江大学 ZHEJIANG UNIVERSITY

锁屏 用户邮件列表 退出 邮件全文搜索

写信

重要邮件
收件箱
待办邮件
已标记邮件
草稿箱
已发送
其他文件夹

收件箱 | 回复: Addit...

回复 | 回复全部 | 转发 | 移动到 | 标记为 | 更多 | 删除

回复: Addition of a new co-author to our revised manuscrip (No.CDDIS-21-3036RR)

石玉华 发送给 sfs@zju.edu.cn

发起会议
2022-01-08 23:27:16

Dear Prof. Shi,

I agree with these changes.

Thanks for your update and have a nice day.

Best regards,

Yuhua Shi

发件人: sfs@zju.edu.cn
发送时间: 2022年1月8日 22:57
收件人: shiyuhua@zju.edu.cn; yyang@zafu.edu.cn; wlxu@zju.edu.cn; shidongyun519@163.com; xuwei88@zju.edu.cn; xyfu@zju.edu.cn; 3150100384@zju.edu.cn; 3170100306@zju.edu.cn
主题: Addition of a new co-author to our revised manuscrip (No.CDDIS-21-3036RR)

Dear all,

I am writing this e-mail to tell you that our authorship have made a change in the last revised Manuscript (No. CDDIS-21-3036R), but I forgot to add the author of Dongyun Shi to the system at that time. She is responsible for the construction of the SYVN1-deficient THP-1 cell. So that I added her name to the system in the final revision (No. CDDIS-21-3036RR). The final authors in our manuscript show as follow: Yuhua Shi#, Yang Yang#, Weilv Xu#, Dongyun Shi, Wei Xu, Xinyu Fu, Qian Lv, Jie Xia, Fushan Shi*.

Please reply if you agree to add her name as a co-author of our article.

If there are any questions, please do not hesitate to contact me.

Best wishes,

Fushan Shi

快捷回复给所有人

搜索一下

13:28
2022/1/9

首页 | 浙江大学邮件系统

https://mail.zju.edu.cn/coremail/XT5/index.jsp?sid=DAYKtZnMaOkQvZnBHNmQfSfGMrXNMiav#mail.read%7C%7B"fid"%3A1%2C"mid"%3A"1%3A1tbiAQIEA1ZdwoWoOgACsY"%2C"mboxa"%3A""%2C"start"%3A7%7D

浙江大学 ZHEJIANG UNIVERSITY

锁屏 用户邮件列表 退出 邮件全文搜索

写信

重要邮件
收件箱
待办邮件
已标记邮件
草稿箱
已发送
其他文件夹

收件箱 | 回复:Additi...

回复 回复全部 转发 移动到 标记为 更多 删除

回复:Addition of a new co-author to our revised manuscrip (No. CDDIS-21-3036RR)

杨杨 发送给 sfs@zju.edu.cn

发起会议 2022-01-08 23:33:37

I agree

Yang Yang

--------------原始邮件--------------
发件人："sfs@zju.edu.cn "<sfs@zju.edu.cn>;
发送时间：2022年1月8日(星期六) 晚上10:57
收件人："shiyuhua" <shiyuhua@zju.edu.cn>;"杨杨" <yyang@zafu.edu.cn>;"wlxu" <wlxu@zju.edu.cn>;"shidongyun519" <shidongyun519@163.com>;"xuwei88" <xuwei88@zju.edu.cn>;"xyfu" <xyfu@zju.edu.cn>;"3150100384" <3150100384@zju.edu.cn>;"3170100306" <3170100306@zju.edu.cn>;
主题：Addition of a new co-author to our revised manuscrip (No. CDDIS-21-3036RR)

Dear all,

I am writing this e-mail to tell you that our authorship have made a change in the last revised Manuscript (No. CDDIS-21-3036R), but I forgot to add the author of Dongyun Shi to the system at that time. She is responsible for the construction of the SYVN1-deficient THP-1 cell. So that I added her name to the system in the final revision (No. CDDIS-21-3036RR). The final authors in our manuscript show as follow: Yuhua Shi#, Yang Yang#, Weilv Xu#, Dongyun Shi, Wei Xu, Xinyu Fu, Qian Lv, Jie Xia, Fushan Shi*.

Please reply if you agree to add her name as a co-author of our article.

If there are any questions, please do not hesitate to contact me.

Best wishes,

Fushan Shi

快捷回复给所有人

记账代理 搜索一下

13:29 2022/1/9

首页 | 浙江大学邮件系统

https://mail.zju.edu.cn/coremail/XT5/index.jsp?sid=DAYKtZnMaOkQvZnBHNmQfSfGMrXNMiav#mail.read%7C%7B"fid"%3A1%2C"mid"%3A"1%3A1tbiAQMEA1ZdwoWweQAGsG"%2C"mboxa"%3A""%2C"start"%3A1%7D

锁屏 用户邮件列表 退出 邮件全文搜索

写信

重要邮件
收件箱
待办邮件
已标记邮件
草稿箱
已发送
其他文件夹

收件箱 | Re: Additio...

回复 | 回复全部 | 转发 | 移动到 | 标记为 | 更多 | 删除

Re: Addition of a new co-author to our revised manuscrip (No. CDDIS-21-3036RR)

徐维律 发送给 sfs@zju.edu.cn

发起会议 2022-01-09 12:18:02

Dear Professor Shi,

I agree.

Best wishes,

Weilv Xu

-----原始邮件-----
发件人:sfs@zju.edu.cn
发送时间:2022-01-08 22:57:47 (星期六)
收件人: shiyuhua@zju.edu.cn, yyang@zafu.edu.cn, wlxu@zju.edu.cn, shidongyun519@163.com, xuwei88@zju.edu.cn, xyfu@zju.edu.cn, 3150100384@zju.edu.cn, 3170100306@zju.edu.cn
抄送:
主题: Addition of a new co-author to our revised manuscrip (No. CDDIS-21-3036RR)

Dear all,

I am writing this e-mail to tell you that our authorship have made a change in the last revised Manuscript (No. CDDIS-21-3036R), but I forgot to add the author of Dongyun Shi to the system at that time. She is responsible for the construction of the SYVN1-deficient THP-1 cell. So that I added her name to the system in the final revision (No. CDDIS-21-3036RR). The final authors in our manuscript show as follow: Yuhua Shi#, Yang Yang#, Weilv Xu#, Dongyun Shi, Wei Xu, Xinyu Fu, Qian Lv, Jie Xia, Fushan Shi*.

Please reply if you agree to add her name as a co-author of our article.

If there are any questions, please do not hesitate to contact me.

Best wishes,

[illegible]

快捷回复给所有人

13:30 2022/1/9

首页 浙江大学邮件系统

https://mail.zju.edu.cn/coremail/XT5/index.jsp?sid=DAYKtZnMaOkQvZnBHNmQfSfGMrXNMiav#mail.read%7C%7B"fid"%3A1%2C"mid"%3A"1%3A1tbiAQMEA1ZdwoWweQADsD"%2C"mboxa"%3A""%2C"start"%3A3%7D

浙江大学 ZHEJIANG UNIVERSITY

锁屏 用户邮件列表 退出 邮件全文搜索

写信

重要邮件
收件箱
待办邮件
已标记邮件
草稿箱
已发送
其他文件夹

收件箱 Re: Additio...

回复 回复全部 转发 移动到 标记为 更多 删除

Re: Addition of a new co-author to our revised manuscrip (No. CDDIS-21-3036RR)

Emily Shi 发送给 sfs@zju.edu.cn

发起会议
2022-01-09 08:55:12

Yes, I agree that, thank you.

Best Regards,
Dongyun Shi

在 2022年1月8日，22:57，sfs@zju.edu.cn 写道：

Dear all,

I am writing this e-mail to tell you that our authorship have made a change in the last revised Manuscript (No. CDDIS-21-3036R), but I forgot to add the author of Dongyun Shi to the system at that time. She is responsible for the construction of the SYVN1-deficient THP-1 cell. So that I added her name to the system in the final revision (No. CDDIS-21-3036RR). The final authors in our manuscript show as follow: Yuhua Shi#, Yang Yang#, Weilv Xu#, Dongyun Shi, Wei Xu, Xinyu Fu, Qian Lv, Jie Xia, Fushan Shi*.

Please reply if you agree to add her name as a co-author of our article.

If there are any questions, please do not hesitate to contact me.

Best wishes,

Fushan Shi

快捷回复给所有人

中 13:30 2022/1/9

首页 | 浙江大学邮件系统

https://mail.zju.edu.cn/coremail/XT5/index.jsp?sid=DAYKtZnMaOkQvZnBHNmQfSfGMrXNMiav#mail.read%7C%7B"fid"%3A1%2C"mid"%3A"1%3A1tbiAQIEA1ZdwoWoOgAEse"%2C"mboxa"%3A""%2C"start"%3A5%7D

浙江大学 ZHEJIANG UNIVERSITY

锁屏 用户邮件列表 退出 邮件全文搜索

写信

重要邮件
收件箱
待办邮件
已标记邮件
草稿箱
已发送
其他文件夹

收件箱 | Re: Additio...

回复 回复全部 转发 移动到 标记为 更多 删除

Re: Addition of a new co-author to our revised manuscrip (No. CDDIS-21-3036RR)

浙大邮箱 发送给 sfs@zju.edu.cn

发起会议
2022-01-08 23:48:13

I agree to add Dongyun Shi as a co-author of our article.
Wei Xu

在 2022年1月8日，22:57，sfs@zju.edu.cn 写道：

Dear all,

I am writing this e-mail to tell you that our authorship have made a change in the last revised Manuscript (No. CDDIS-21-3036R), but I forgot to add the author of Dongyun Shi to the system at that time. She is responsible for the construction of the SYVN1-deficient THP-1 cell. So that I added her name to the system in the final revision (No. CDDIS-21-3036RR). The final authors in our manuscript show as follow: Yuhua Shi#, Yang Yang#, Weilv Xu#, Dongyun Shi, Wei Xu, Xinyu Fu, Qian Lv, Jie Xia, Fushan Shi*.

Please reply if you agree to add her name as a co-author of our article.

If there are any questions, please do not hesitate to contact me.

Best wishes,

Fushan Shi

快捷回复给所有人

张拉膜结构 搜索一下

13:31
2022/1/9

首页 | 浙江大学邮件系统

https://mail.zju.edu.cn/coremail/XT5/index.jsp?sid=EAXOoLCOCDbGRQIZEuyFwuTCiEugEGFT#mail.read%7C%7B"fid"%3A1%2C"mid"%3A"1%3A1tbiAQMEA1ZdwoWweQAMsM"%2C"mboxa"%3A""%2C"start"%3A0%7D

浙江大学 ZHEJIANG UNIVERSITY

锁屏 用户邮件列表 退出 邮件全文搜索

写信

重要邮件
收件箱
待办邮件
已标记邮件
草稿箱
已发送
其他文件夹
已删除
垃圾邮件
病毒文件夹
Notes

收件箱 | Stork has ... | Re: Additio...

回复 回复全部 转发 移动到 标记为 更多 删除

Re: Addition of a new co-author to our revised manuscrip (No.CDDIS-21-3036RR)

xyfu 发送给 sfs

发起会议
2022-01-09 15:59:34

Yes, I agree.

Xinyu Fu

发自我的iPhone

------------------ Original ------------------

From: sfs <sfs@zju.edu.cn>
Date: 周六, 1月 8, 2022 22:57
To: shiyuhua <shiyuhua@zju.edu.cn>, yyang <yyang@zafu.edu.cn>, wlxu <wlxu@zju.edu.cn>, shidongyun519 <shidongyun519@163.com>, xuwei88 <xuwei88@zju.edu.cn>, xyfu <xyfu@zju.edu.cn>, 3150100384 <3150100384@zju.edu.cn>, 3170100306 <3170100306@zju.edu.cn>
Subject: Re: Addition of a new co-author to our revised manuscrip (No.CDDIS-21-3036RR)

Dear all,

I am writing this e-mail to tell you that our authorship have made a change in the last revised Manuscript (No. CDDIS-21-3036R), but I forgot to add the author of Dongyun Shi to the system at that time. She is responsible for the construction of the SYVN1-deficient THP-1 cell. So that I added her name to the system in the final revision (No. CDDIS-21-3036RR). The final authors in our manuscript show as follow: Yuhua Shi#, Yang Yang#, Weilv Xu#, Dongyun Shi, Wei Xu, Xinyu Fu, Qian Lv, Jie Xia, Fushan Shi*.

Please reply if you agree to add her name as a co-author of our article.

If there are any questions, please do not hesitate to contact me.

Best wishes,

Fushan Shi

快捷回复给所有人

瓦伦炮轰皇马和裁判 搜索一下

16:34
2022/1/9

首页 浙江大学邮件系统

https://mail.zju.edu.cn/coremail/XT5/index.jsp?sid=DAYKtZnMaOkQvZnBHNmQfSfGMrXNMiav#mail.read%7C%7B"fid"%3A1%2C"mid"%3A"1%3A1tbiAQMEA1ZdwoWweQAHsH"%2C"mboxa"%3A""%2C"start"%3A0%7D

浙江大学 ZHEJIANG UNIVERSITY　锁屏　用户邮件列表　退出　邮件全文搜索

写信　重要邮件　收件箱　待办邮件　已标记邮件　草稿箱　已发送　其他文件夹

收件箱　Re: Additio...

回复　回复全部　转发　移动到　标记为　更多　删除

Re: Addition of a new co-author to our revised manuscrip (No. CDDIS-21-3036RR)

3150100384 发送给 sfs@zju.edu.cn　　2022-01-09 12:36:57　发起会议

Dear Mr. Shi,

Thank you for your email. I agree with the amendments to the article.

Best regards.

Qian Lv

---- Replied Message ----

From	sfs@zju.edu.cn<sfs@zju.edu.cn>
Date	01/08/2022 22:57
To	shiyuhua@zju.edu.cn<shiyuhua@zju.edu.cn>,<br>yyang@zafu.edu.cn<yyang@zafu.edu.cn>,<br>wlxu@zju.edu.cn<wlxu@zju.edu.cn>,<br>shidongyun519@163.com<shidongyun519@163.com>,<br>xuwei88@zju.edu.cn<xuwei88@zju.edu.cn>,<br>xyfu@zju.edu.cn<xyfu@zju.edu.cn>,<br>3150100384@zju.edu.cn<3150100384@zju.edu.cn>,<br>3170100306@zju.edu.cn<3170100306@zju.edu.cn>
Subject	Addition of a new co-author to our revised manuscrip (No. CDDIS-21-3036RR)

Dear all,

I am writing this e-mail to tell you that our authorship have made a change in the last revised Manuscript (No. CDDIS-21-3036R), but I forgot to add the author of Dongyun Shi to the system at that time. She is responsible for the construction of the SYVN1-deficient THP-1 cell. So that I added her name to the system in the final revision (No. CDDIS-21-3036RR). The final authors in our manuscript show as follow: Yuhua Shi#, Yang Yang#, Weilv Xu#, Dongyun Shi, Wei Xu, Xinyu Fu, Qian Lv, Jie Xia, Fushan Shi*.

Please reply if you agree to add her name as a co-author of our article.

If there are any questions, please do not hesitate to contact me.

Best wishes,

快捷回复给所有人

张拉膜结构　搜索一下　13:32 2022/1/9

首页　浙江大学邮件系统

https://mail.zju.edu.cn/coremail/XT5/index.jsp?sid=DAYKtZnMaOkQvZnBHNmQfSfGMrXNMiav#mail.read%7C%7B"fid"%3A1%2C"mid"%3A"1%3A1tbiAQMEA1ZdwoWweQACsC"%2C"mboxa"%3A""%2C"start"%3A4%7D

浙江大学 ZHEJIANG UNIVERSITY

锁屏　用户邮件列表　退出　邮件全文搜索

写信

重要邮件
收件箱
待办邮件
已标记邮件
草稿箱
已发送
其他文件夹

收件箱　Re: Additio...

回复　回复全部　转发　移动到　标记为　更多　删除

Re: Addition of a new co-author to our revised manuscrip (No. CDDIS-21-3036RR)

3170100306 发送给 sfs@zju.edu.cn

发起会议
2022-01-09 07:16:20

I agree, Jie Xia

---- Replied Message ----

From　sfs@zju.edu.cn<sfs@zju.edu.cn>
Date　01/08/2022 22:57
To　shiyuhua@zju.edu.cn<shiyuhua@zju.edu.cn>,
yyang@zafu.edu.cn<yyang@zafu.edu.cn>,
wlxu@zju.edu.cn<wlxu@zju.edu.cn>,
shidongyun519@163.com<shidongyun519@163.com>,
xuwei88@zju.edu.cn<xuwei88@zju.edu.cn>,
xyfu@zju.edu.cn<xyfu@zju.edu.cn>,
3150100384@zju.edu.cn<3150100384@zju.edu.cn>,
3170100306@zju.edu.cn<3170100306@zju.edu.cn>
Subject　Addition of a new co-author to our revised manuscrip (No. CDDIS-21-3036RR)

Dear all,

I am writing this e-mail to tell you that our authorship have made a change in the last revised Manuscript (No. CDDIS-21-3036R), but I forgot to add the author of Dongyun Shi to the system at that time. She is responsible for the construction of the SYVN1-deficient THP-1 cell. So that I added her name to the system in the final revision (No. CDDIS-21-3036RR). The final authors in our manuscript show as follow: Yuhua Shi#, Yang Yang#, Weilv Xu#, Dongyun Shi, Wei Xu, Xinyu Fu, Qian Lv, Jie Xia, Fushan Shi*.

Please reply if you agree to add her name as a co-author of our article.

If there are any questions, please do not hesitate to contact me.

Best wishes,

Fushan Shi

快捷回复给所有人

粉碎机厂家　搜索一下

13:32
2022/1/9
